# Oral Administration of a Fusion Protein between the Cholera Toxin B Subunit and the 42-Amino Acid Isoform of Amyloid-β Peptide Produced in Silkworm Pupae Protects against Alzheimer's Disease in Mice

**DOI:** 10.1371/journal.pone.0113585

**Published:** 2014-12-03

**Authors:** Si Li, Zhen Wei, Jian Chen, Yanhong Chen, Zhengbing Lv, Wei Yu, Qiaohong Meng, Yongfeng Jin

**Affiliations:** 1 Institute of Biochemistry, College of Life Sciences, Zhejiang University, Hangzhou, China; 2 Institute of Biochemistry, College of Life Sciences, Zhejiang Sci-Tech University, Hangzhou, China; 3 Laboratory Animal Center of Zhejiang University, Zhejiang University, Hangzhou, China; 4 Institute of Aging Research, School of Medicine, Hangzhou Normal University, Hangzhou, China; University of Akron, United States of America

## Abstract

A key molecule in the pathogenesis of Alzheimer's disease (AD) is a 42-amino acid isoform of the amyloid-β peptide (Aβ42), which is the most toxic element of senile plaques. In this study, to develop an edible, safe, low-cost vaccine for AD, a cholera toxin B subunit (CTB)-Aβ42 fusion protein was successfully expressed in silkworm pupae. We tested the silkworm pupae-derived oral vaccination containing CTB-Aβ42 in a transgenic mouse model of AD. Anti-Aβ42 antibodies were induced in these mice, leading to a decreased Aβ deposition in the brain. We also found that the oral administration of the silk worm pupae vaccine improved the memory and cognition of mice, as assessed using a water maze test. These results suggest that the new edible CTB-Aβ42 silkworm pupae-derived vaccine has potential clinical application in the prevention of AD.

## Introduction

The main neuropathological feature of Alzheimer's disease (AD) is excessive extracellular β-amyloid protein (Aβ) deposition that forms senile plaques. Aβ42 aggregates are the most toxic components of senile plaques, which are crucial in the pathogenesis of AD. The deposition of Aβ in the brain is a principal target in many AD treatment strategies [1], [2]. A current hot topic in the field is using immunotherapy to reduce and eliminate Aβ deposition. The concomitant reduced β-amyloid burden is associated with restored cognitive function. The Aβ vaccine has been shown to decrease and eliminate Aβ deposition in the brains of AD transgenic mice and to impair behavioral and cognitive disorders in experimental mice [3], [4]. A phase II clinical trial on the Aβ injection vaccine AN1792, produced by the ELAN corporation, was initiated, but the trial was stopped prematurely because aseptic meningitis occurred in 6% of trial subjects [5], [6]. This reaction is related to the induction of TH1-type responses. As a result, a vaccine producing a mild antigen-antibody reaction and/or an appropriate immunological adjuvant resulting in a decreased TH1 immune response and an increased TH2 immune response is needed for the prevention and treatment of AD.

As a “bioreactor” for the production of Aβ proteins, the silkworm is a good choice. The silkworm *Bombyx mori* is most well known for its use in silk production. Silkworm pupae are a low-cost byproduct of the silk reeling industry that has potential for high-capacity scaling to agricultural levels. In recent years, the Chinese health department has listed silkworm pupae in the category of "new food raw material originating from traditional food" [7]. The “silkworm bioreactor” has several other advantages. It is safe for vertebral animals [8], and because it is a eukaryotic expression system, the proteins expressed in silkworms obtain post-translational modifications such as phosphorylation, glycosylation and disulfide bonds [9]. In addition, the proteins created for oral consumption are more stable in the stomach and intestines because of the protease inhibitors and biocapsule-like fat in silkworms [10]. The “silkworm bioreactor” is unquestionably an ideal expression system for producing oral vaccines, although edible silkworm vaccines may induce low immune responses because of low expression levels of target antigens. A strategy to overcome this obstacle is to increase antigen uptake into mucosal immune systems by using CTB as a carrier for the fused antigen.

CTB is a non-toxic portion of cholera toxin that is composed of five identical polypeptides (11.5 kDa), and it assembles into a highly stable pentameric ring structure in bacteria. CTB functions as an effective carrier molecule for fused foreign proteins [11], and it is a non-TH1-inducing adjuvant [12] that has immunomodulating effects. It lowers the threshold concentration of the conjugated antigen required for immune cell activation and increases CD40 and CD86 expression on antigen-presenting cells (APCs) [13].

In this study, we expressed the CTB-Aβ42 fusion gene in silkworm pupae. We found that the production level of the expressed fusion protein reached 0.5 µg/g, and the recombinant protein was able to form pentamers. To determine whether silkworm pupae-derived CTB-Aβ42 has prophylactic effects in AD, B6C3-Tg (APPswe, PSEN1dE9) 85Dbo/Mmjax mice were immunized orally. The AD model mice showed an elevated Aβ antibody titer and reduced levels of Aβ in the brain. Cognitive function was assessed using a water maze test. The aim of this study was to lay the foundation for development of a useful, safe system for use in an oral vaccination protocol against Alzheimer's disease.

## Materials and Methods

### Reagents, vector, *E*. *coli*, cells and mice

*E*. *coli* TG1, the transfer vector pBacPAK_8_, linearized *Bombyx mori* baculovirus Bm-BacPAK6 and BmN cells were from the collections of the College of Life Sciences of Zhejiang Sci-Tech University (China). Rabbit anti-human CT polyclonal antibodies, standard CTB and monosialoganglioside-GM1 were purchased from Sigma-Aldrich (USA); mouse anti-human Aβ_x-42_ antibodies were purchased from Millipore (USA); and standard Aβ42 was purchased from GenScript (USA). Horseradish peroxidase goat anti-rabbit and goat anti-mouse antibodies were obtained from Dingguo Biotech (China). Restriction endonucleases, Taq polymerase, T4 DNA ligase and the DNA marker were obtained from TaKaRa Biotech (Japan). The protein marker was produced by MBI Fermentas (Canada). Protease K and X-gal were purchased from Roche Diagnostics (Switzerland). TC-100 insect medium and fetal bovine serum were purchased from Gibco BRL (USA). The cell lysis buffer was purchased from Beyotime Biotechnology Company (China). The 96-well microtiter plates were purchased from COSTAR (USA). Male B6C3-Tg(APPswe, PSEN1dE9) transgenic mice (Permit Number: SCXK(苏)2010-0001) carrying the K595N and M596L mutations in APP (a model of AD) were acquired from the Nanjing Biomedical Research Institute of Nanjing University (China). Fifth-instar silkworm pupae (Jingsong × Haoyue) were purchased from Zhongqi Pharmaceutical Company (China). This study was performed in strict accordance with the recommendations in the Guide for the Care and Use of Laboratory Animals of the National Institutes of Health. The protocol was approved by the Committee on the Ethics of Animal Experiments of the medical department of Zhejiang University (Permit Number: ZJU201307-1-01-067). All surgeries were performed under sodium pentobarbital anesthesia, and all efforts were made to minimize animal suffering.

### Construction of recombinant baculovirus BmNPV-CTB–Aβ42

Four synthetic oligonucleotides encoding the human Aβ42 were designed to amplify the Aβ42 gene by recursive PCR (Table 1) [14]. Prior to construction of the CTB-Aβ42 fusion gene, three primers were designed: AD-CTB-2: 5'-**GGGGCCGGGGCC**ATTTGCCATACTAATTG-3, AD-CTB-Aβ-1: 5'-**GGCCCCGGCCCC**GATGCAGAATTCCGACA-3 and P3: 5'- CG*GGATCC*ATGATTAAATTAAAATTTGG-3' (*Bam*HI). The oligonucleotide sequence in bold type encodes a flexible hinge tetrapeptide (GPGP) fused to the 3′-end of the CTB gene and the 5′-end of the Aβ42 gene. To construct the CTB-GPGP gene fragment, primers P3 and AD-CTB-2 were used as the forward and reverse primers, respectively. Similarly, primers AD-CTB-Aβ-1 and AD-R1 (Table 1) were used to construct the GPGP–Aβ42 gene fragment. Based on the overlap region (GPGP), CTB–GPGP and GPGP–Aβ42 fragments were amplified with the 5′-primer of the CTB gene (P3) and the 3′-primer of the Aβ42 gene (AD-R1) by PCR. The CTB–Aβ42 fusion gene was inserted into the transfer vector pBacPAK_8_ downstream of the polyhedrin promoter. The recombinant transfer vector pBacPAK_8_-CTB-Aβ42 was cotransfected into BmN cells with linearized Bm-BacPAK6 virus DNA that was digested by the restriction enzyme *Bsu*36I. The recombinant virus was screened, plaque-purified, and identified by sequencing. Pure recombinant virus was obtained and was designated BmNPV-CTB–Aβ42. The Reed-Muench method was used to calculate the recombinant virus titer [15].

**Table 1:** Four oligonucleotides for the construction of the Aβ42 gene.

No.	Sequence	Length
AD-F1	5'-GG***GGATCC***GATGCAGAATTCCGACATGACTCAGGATATGAAGTTCATCA-3	49
AD-F2	5'-GATATGAAGTTCATCATCAAAAATTGGTGTTCTTTGCAGAAGATGTGGGT-3'	50
AD-R1	5'-GG***CTCGAG***CTACGCTATGACAACACCGCCCACCATGAGTCCAATGATTG-3	49
AD-R2	5'-ATGAGTCCAATGATTGCACCTTTGTTTGAACCCACATCTTCTGCAAAGAA-3	50

The italics in AD-F1 and AD-R1 indicate BamHI and EcoRI restriction endonuclease sites, respectively.

### Expression of recombinant CTB- Aβ42 protein in BmN cells and silkworm pupae

BmN cells (2×10^6^ cells/flask) and silkworm pupae were infected with the recombinant virus BmNPV-CTB–Aβ42 (MOI  = 10). The cells were harvested daily by centrifugation at 3000 r/min for 5 min and incubated in cell lysis buffer on ice. The lysed cells were centrifuged at 12,000 r/min for 10 min, and the supernatant was collected. Silkworm pupae were infected with the recombinant virus BmNPV-CTB–Aβ42 by subcutaneous injection at approximately 2×10^5^ particles per pupae in a volume of 0.005 ml. The silkworm pupae were collected from the second to the seventh day for ELISA and western blot assays. The collected pupae were homogenized into powder by biological cryo-freeze-drying technology.

### SDS-PAGE and western blotting assay

The BmN cells and silkworm pupae powder samples described above were diluted in PBS and centrifuged at 12,000 r/min for 10 min. All of the supernatant samples were either boiled for 5 min prior to electrophoresis or loaded directly onto the gel without heat treatment. Next, the samples were electrophoresed on a 12% SDS-PAGE gel for 60 min at 100 V in Tris-glycine buffer (25 mM Tris, 250 mM glycine, pH 8.3, 0.1% SDS) to detect the presence of fusion proteins. The separated proteins were transferred from the gel to a PVDF membrane (Millipore) by electroblotting on an AE-6675 blotter (ATTO Corp) at 2 mA/cm^2^ for 90 min. The membrane was incubated in TBS buffer (10 mM Tris·Cl, pH 7.4, 0.15 M NaCl) for 10 min at room temperature with gentle shaking and then blocking buffer (3% BSA added to 1× TBS) for 2 h at 37°C with gentle shaking. The membrane was incubated in a 1∶4000 dilution of mouse anti-Aβ_x-42_ monoclonal antibody at 37°C for 1.5 h, followed by three washes with TBST (0.05% Tween-20 added to 1× TBS). Next, the membrane was incubated in a 1∶1000 dilution of goat anti-rat IgG antibody conjugated with horseradish peroxidase for 1 h at 37°C and washed three times with TBST. Detection was performed using an ECL kit.

#### ELISA and GM1-ELISA binding assay

The CTB-Aβ42 fusion protein levels in BmN cells and silkworm pupae were determined using a quantitative ELISA assay with serial dilutions. BmN cells and silkworm pupae inoculated with linearized Bm-BacPAK_6_ virus were used as a negative control. The collected cells were suspended in 0.8 ml PBS, lysed in 0.2 ml of cell lysis buffer and then centrifuged at 12,000×g for 10 min at 4°C to collect the supernatant. The sampled pupae were homogenized, and the homogenate was centrifuged at 12,000×*g* for 20 min at 4°C. The resulting supernatant from each sample was diluted 1∶100 and analyzed by ELISA. A 96-well microtiter plate was loaded with the supernatant from cell and silkworm pupae samples, a CTB standard protein sample and PBS in bicarbonate buffer (15 mmol/l NaCO3, 35 mmol/l NaHCO3, pH 9.6) and incubated for 18 hours at 4°C. The plate was washed three times in PBS containing 0.05% Tween-20 (PBST). The plate was blocked in 1% bovine serum albumin (BSA) in PBS (100 µl/well) for 2 h at 37°C, followed by three washes with PBST. The plate was incubated with a 1∶8000 dilution of rabbit anti-cholera toxin primary antibody (100 µl/well) at 37°C for 2 h, followed by three washes with PBST. The plate was next incubated with a 1∶1000 dilution of anti-rabbit IgG conjugated with horseradish peroxidase (100 µl/well) for 2 h at 37°C and washed three times with PBST. Finally, the chromogenic substrate *O-*phenylenediamine was added to each well (100 µl/well, pH 5.0). The plate was incubated for 15 min at 37°C to develop the color, and then 2 M H_2_SO_4_ (50 µl/well) was added to stop the reaction. After the plate was cooled to room temperature, the absorbance of each well was measured at 490 nm using a Labsystems Multiskan MS ELISA plate reader (SPECTRAmax plus 384, Molecular Devices, USA).

A GM1-ELISA was performed to detect the affinity of the silkworm-derived CTB-Aβ42 fusion protein for GM1-ganglioside. The microtiter plate was coated with monosialoganglioside (GM1) by incubating the plate with 10 µg/ml GM1 (50 µl/well) in methanol at 4°C overnight. The wells were next coated with 1% BSA (100 µl/well). Identical dilutions of linearized baculovirus silkworm pupae and bacterial CTB (Sigma) samples were used as negative and positive controls, respectively. The remainder of the procedure was identical to the ELISA assay described above. The ELISA and GM1-ELISA assays were performed using the methods of Zhaohui Gong et al. [10].

### Preparation of silkworm pupae powder and mouse immunizations

The silkworm pupae that expressed the CTB-Aβ42 fusion protein were made into powder by biological cryo-freeze-drying technology. The silkworm pupae powder was dissolved in PBS and centrifuged at 10,000 r/min for 10 min, and the supernatant was then delivered to the mice by gastric intubation. The quantity of silkworm pupae powder administered to each mouse was adjusted to provide a dose of 6 µg of Aβ42.

Twenty-one male B6C3-Tg (APPswe, PSEN1dE9) transgenic mice (5 wks old) were randomly divided into three groups of seven mice each: control, Aβ42 and CTB-Aβ42. Control mice were orally administered PBS. The Aβ42 group of mice was immunized with silkworm pupae expressing the Aβ42 protein, and the CTB-Aβ42 group of mice was immunized with silkworm pupae expressing the CTB-Aβ42 fusion protein. All of the mice received a dose of the vaccine every day from 1 to 10 months of age. On the 11th month, whole blood was collected from their hearts. All of the mice were housed 3–4 to a cage and maintained with ad libitum food and water.

### Analysis of anti-Aβ42 antibodies in the serum of mice

The collected blood was stored at 37°C for 3 h and then centrifuged at 4°C for 20 min at 2500 r/min before the serum was extracted. The antibody titers against Aβ42 in the serum were detected by indirect ELISA [10]. Aβ42 standard protein was used as the well-coating antigen (300 ng/well), and serial dilutions of pooled sera were added to the coated microtiter plate wells. Horseradish peroxidase-conjugated anti-rat IgG1 antibodies were used as secondary antibodies. The previously described ELISA protocol was followed for the remaining washes and final determination of the A450 value.

### Water maze test

Water maze tests were performed at the end of 10 months. The water maze apparatus (Zhenghua Huaibei, China) consisted of a circular tank (1.2 m in diameter and 0.50 m high) manufactured in China. The tank was filled with water (23–24°C, made opaque with 60 ml of prepared Chinese black ink) to a depth of 30 cm and was divided into four quadrants: north, south, east and west. Each quadrant had an equally spaced white graphic marker (triangle, square, diamond and circular). An escape platform (made of organic glass 8 cm in diameter) was submerged 2 cm below the water's surface in the center of one of the quadrants. The movements of the animal in the tank were recorded using a video camera above the center of the tank. A shading cloth was hung 150 cm above the pool.

#### Procedure

First, the mice were pre-trained to assess their swimming abilities. Each mouse was allowed to swim freely for 90 s without the escape platform. Next, they were released to swim in a 15-cm-wide alley to reach the platform. The test program included the following. (1) A navigation test performed for 5 consecutive days with three trials per day. The mice were released to find and climb onto the hidden platform in the tank within 90 s. If a mouse failed to find a platform within 90 s, it was guided to find the platform and rest on the platform for 20 s. Each mouse was tested four times at different platform quadrants and was given 30 min of rest between trials. Escape latency (s), length of swim distance (cm), and swim speed (cm/s) were recorded. (2) A probe trial test to assess memory was conducted on the sixth day. The platform was removed from the pool after the last navigation trial and the mice were released in every quadrant and given 90 s to swim as usual. The percentage of time spent in the target quadrant (where the hidden platform used to be) was recorded, and the number of passes through the platform area was analyzed.

### Immunostaining of mouse brain slices and image analysis

After the water maze tests, all of the mice were sacrificed. One brain hemisphere was fixed in 10% formaldehyde for 24 h at room temperature and then embedded in paraffin. The paraffin-embedded tissues were cut into 4-µm sagittal sections using a MICROM frozen microtome (HM325,Germany). Sections were immunostained using the primary rabbit polyclonal anti-beta amyloid antibody (Abcam, Cambridge, UK) and diaminobenzidine (DAB). After staining, the percent area occupied by immunoreactive deposits for Aβ in brain was quantified using Image Analyzer software (ImageJ, USA). A monochromatic base threshold was set to distinguish nonspecific staining, and pixels corresponding to immunolabeled structures were selected. Six equidistant sagittal sections were evaluated for each mouse. Image acquisition was performed in a single session using an Olympus BX61 camera (Tokyo, Japan), and the threshold of detection was held constant during analysis.

#### ELISA for brain Aβ

One frozen cerebral hemisphere was homogenized in TBS and then centrifuged at 200,000×g for 20 min at 4°C. The pellet was homogenized with Guanidine-Tris buffer (5.0 M guanidine HCl/50 mM Tris) and sonicated for 30 s, and then the sample was incubated at room temperature for 1 hour and centrifuged at 100,000×g for 30 min at 4°C. The precipitate contained the insoluble fraction. We measured the amount of Aβ42 in each brain using a sandwich ELISA method.

#### Statistical analysis

The statistical analysis was performed using SPSS software (version 19, IBM Company, USA). The data are presented as the means ± SE. Statistical comparisons were performed using ANOVA analyses for testing the significance, and a P value of less than 0.05 was considered statistically significant (<0.01 very significant).

## Results

### Construction and identification of the recombinant baculovirus BmNPV-CTB- Aβ42

**Figure 1:**
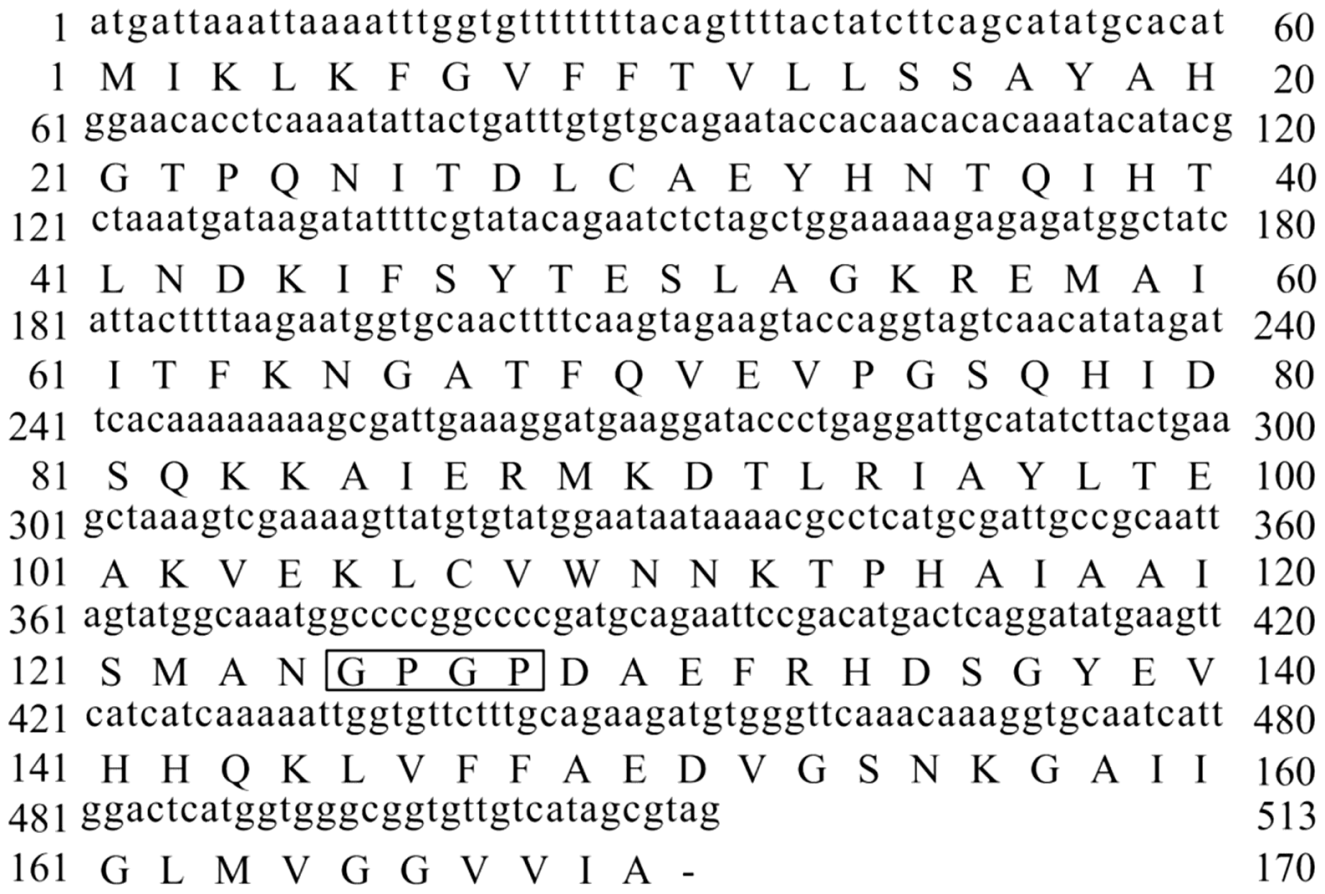
The gene and protein sequences of CTB- Aβ42. The box contains the amino acid GPGP linking CTB and the Aβ42 gene.

**Figure 2:**
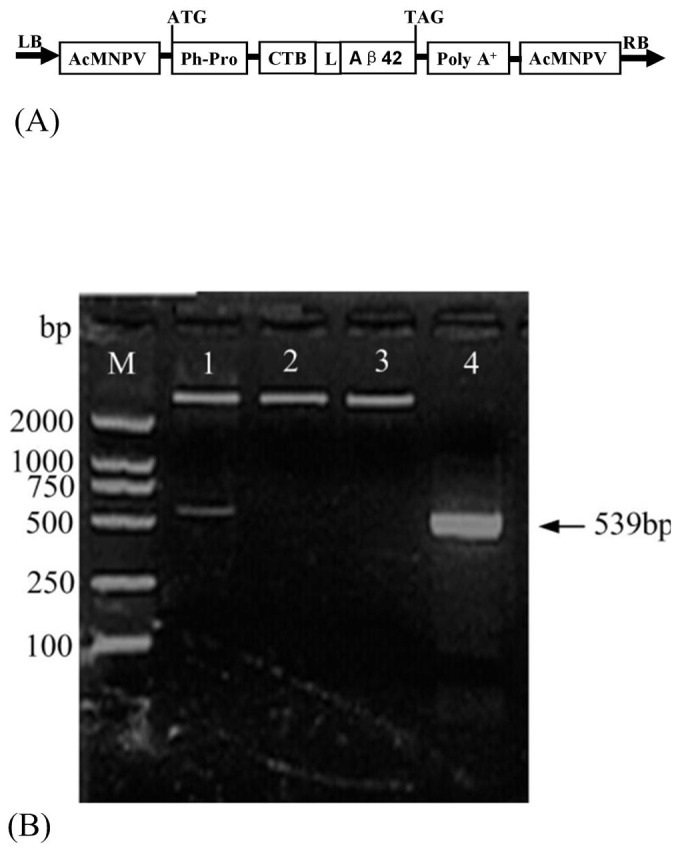
Schematic structure(A), restriction enzyme digestion and PCR identification(B) of recombinant plasmid pBacPAK_8_-CTB-Aβ42. (**A**) LB, left border; AcMNPV, *Autographa Californica* Nuclear Polyhedrosis Virus; Ph-Pro, AcMNPV polyhedrin promoter; L, linker peptide (GPGP); poly A^+^, polyadenylation signal; RB, right border. (**B**) M, DNA marker DL 2000 (TakaRa); lane 1, recombinant plasmid digested with *Bam*HI and *Xho* I; lane 2, recombinant plasmid digested with *Bam*HI; lane 3, recombinant plasmid digested with *Xho* I; lane 4, PCR amplification of recombinant plasmid.

The TP-PCR method was used to anchor the gene fragment encoding Aβ42 at the 3′-end of the CTB gene to form a fusion gene. To support the natural conformations of CTB and Aβ42, a flexible hinge tetrapeptide (GPGP) was introduced between the two peptides. The full-length nucleotide and deduced amino acid sequences are shown in Fig. 1. The PCR product was purified and digested with *Bam*HI and *Xho*I and was subcloned into the expression vector pBacPAK_8_. The results of the double restriction endonuclease enzyme digestion and PCR amplification sequencing (data not shown) confirmed the correct insertion of the fusion gene encoding CTB–Aβ42 into the pBacPAK_8_ vector (Fig. 2A, B).

The recombinant plasmid pBacPAK_8_-CTB-Aβ42 and linearized BmNPV DNA digested with *Bsu*36I were co-transfected in BmN cells. After three rounds of plaque screening and purification, recombinant virus BmNPV-CTB-Aβ42 was obtained. The recombinant viral genomic DNA was extracted from the infected BmN cells and sequenced to confirm that the CTB- Aβ42 gene was correctly inserted into the baculovirus genome under control of the polyhedrin promoter.

According to the Reed-Muench formula [15], the dilution of the recombinant virus was 2.5×10^8^ pfu/ml.

### Expression and identification of recombinant CTB-Aβ42 protein in silkworm pupae

#### ELISA analysis

**Figure 3:**
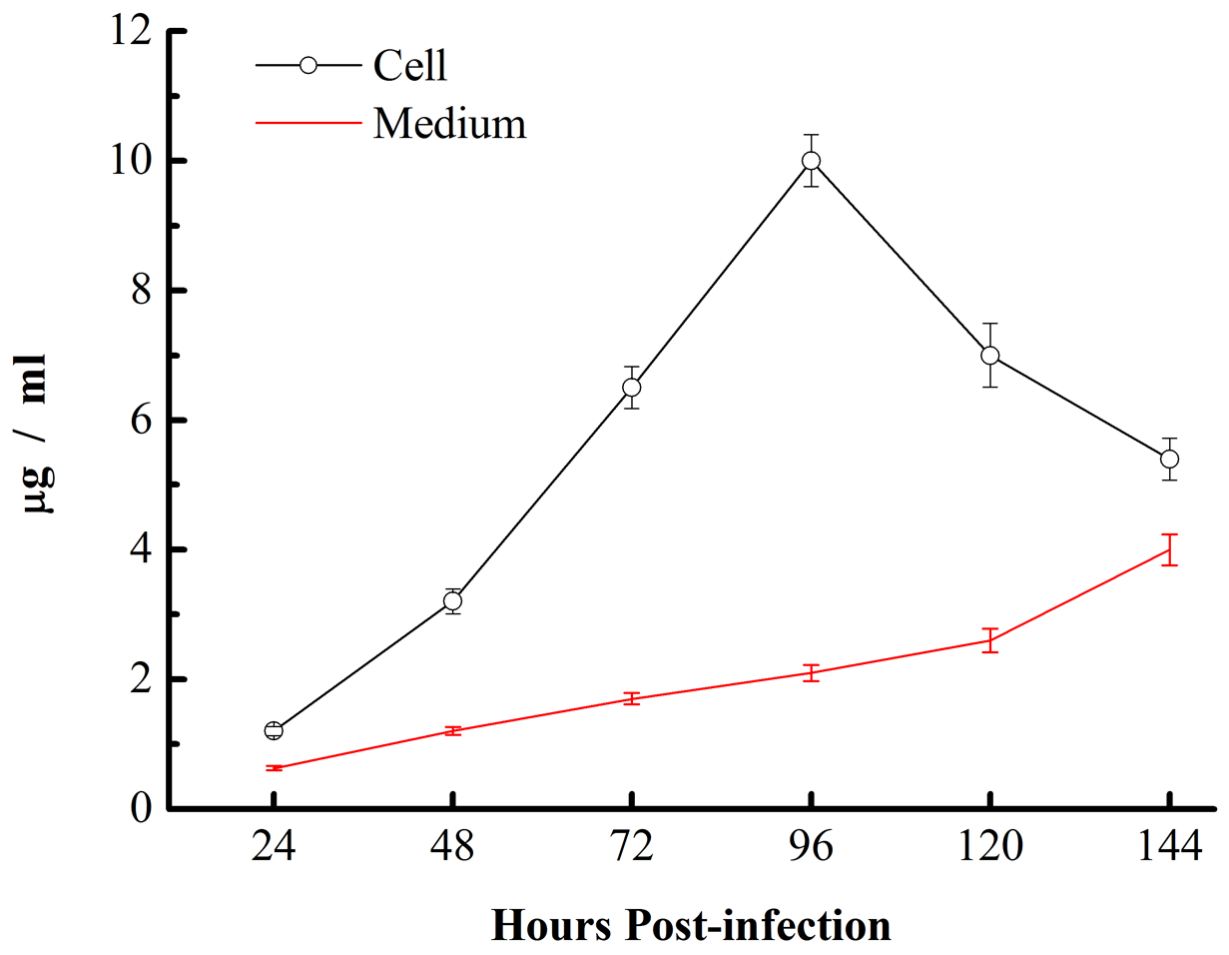
Quantitative analysis of protein production levels in BmN cells. Data are presented as the mean concentrations.

The silkworm BmN cells developed symptoms of infection that were observed under a microscope; cells became round on approximately the third day and then ruptured, releasing virus particles into the culture medium on approximately the fifth to the seventh day. To evaluate the expression of CTB-Aβ42 in the cells, intracellular and extracellular samples of the inoculated silkworm BmN cells were collected on different days. The CTB standard protein was used to make a standard curve, and the samples were diluted with the coating liquid to the appropriate concentration for ELISA testing. The highest detectable level of fusion protein in cells was 10 µg/2×10^6^ cells at the fourth day post-infection. During the late phase of infection, the infected cells began to lyse, and the recombinant fusion protein was released into the culture medium. The maximum concentration of the recombinant protein recovered from the culture medium reached 4 µg/ml (Fig. 3).

**Figure 4:**
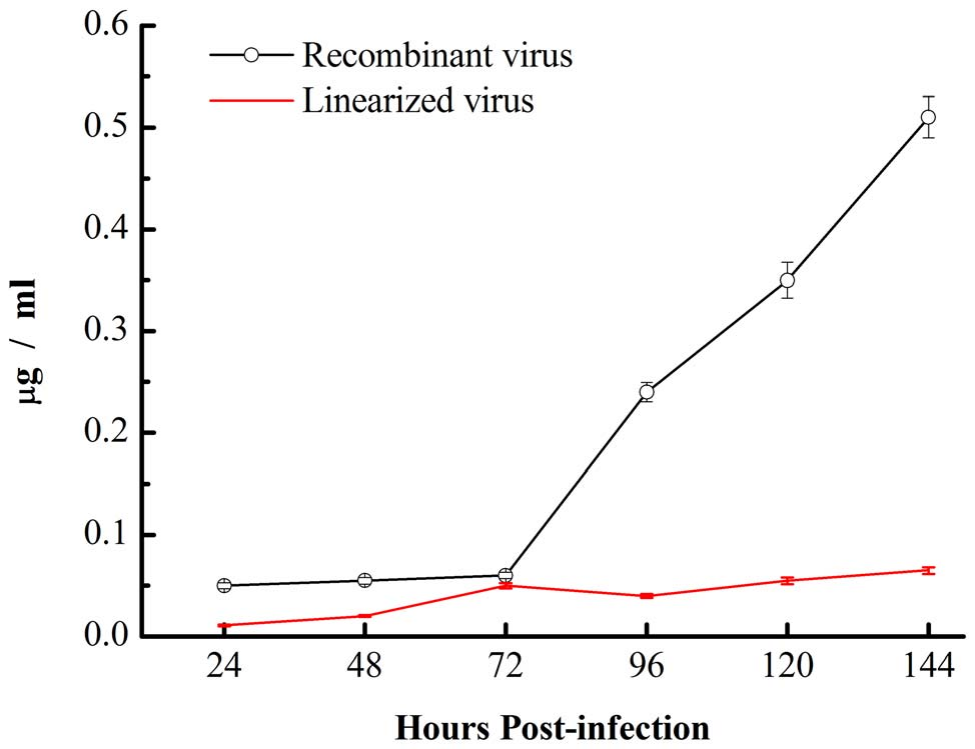
Quantitative analysis of protein production levels in silkworm pupae. Data are presented as the mean concentrations.

To evaluate the expression of CTB-Aβ42, silkworm pupae were subcutaneously injected with recombinant virus BmNPV-CTB-Aβ42. Four days after infection, the surface of silkworm pupae became brown and softened gradually. By the sixth day, some of the pupae bodies were close to rupture. To analyze the amount of CTB-Aβ42 expressed in silkworm pupae, silkworm pupae were randomly sampled at 24 h intervals after infection. The results (Fig. 4) show that the production of recombinant protein increased continuously with increasing infection days. At the sixth day post-infection, the maximum amount of fusion protein reached 0.5 µg/g in the silkworm pupae.

#### Western blot analysis

**Figure 5:**
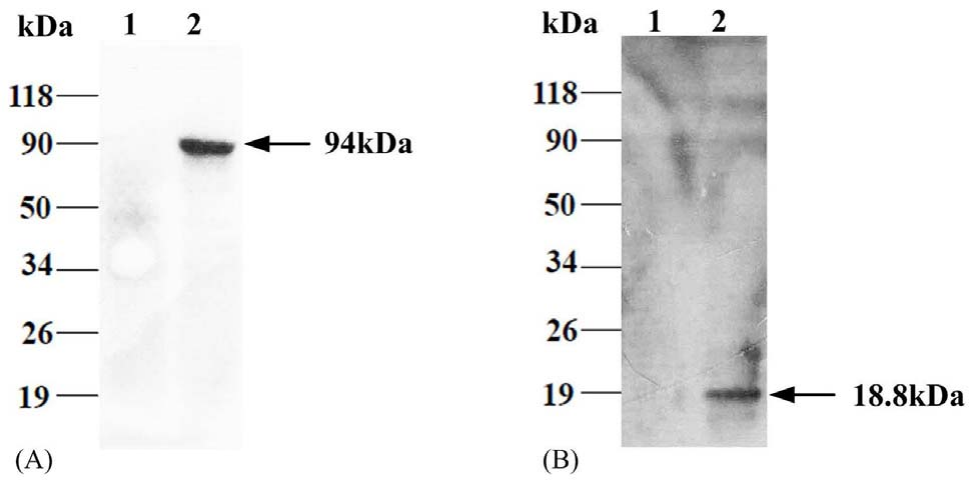
Western blot analysis of the CTB-Aβ42 fusion protein expressed silkworm pupae. (**A**) Lanes 1: unboiled silkworm pupa sample infected with linearized virus, Lanes 2: unboiled silkworm pupa sample infected with recombinant virus; (**B**) Lanes 1: boiled silkworm pupa sample infected with linearized virus, Lanes 2: boiled silkworm pupa sample infected with recombinant virus.

On the sixth day post-infection, the proteins from silkworm pupae infected with recombinant and linearized virus were separated by 12% SDS-PAGE gels and assayed by western blot. To detect potential pentamers and monomers of CTB-Aβ42 recombinant protein, the silkworm pupae proteins were examined using both boiled and unboiled samples. Western blot analysis showed that the CTB-Aβ42 fusion protein from silkworm pupae adopted a pentameric conformation, whereas the heat-treated fusion protein was only detected as monomers. The western blotting indicated that the recombinant CTB-Aβ42 protein was approximately 19 kD in monomers and 94 kD in pentamers, and these forms reacted with anti-Aβ42 antibodies. No signal was observed in silkworm pupae infected with linearized virus. This analysis indicates that CTB-Aβ42 was expressed in silkworm pupae (Fig. 5).

### Pentameric formation analysis of CTB-Aβ42

CTB's efficacy as a mucosal carrier is believed to be associated with its strong ability to bind monosialoganglioside (GM1). The five identical CTB monomers are arranged in a ring-like pentameric configuration, with each having a single binding site for the GM1 receptor present on most nucleated cells.

**Figure 6:**
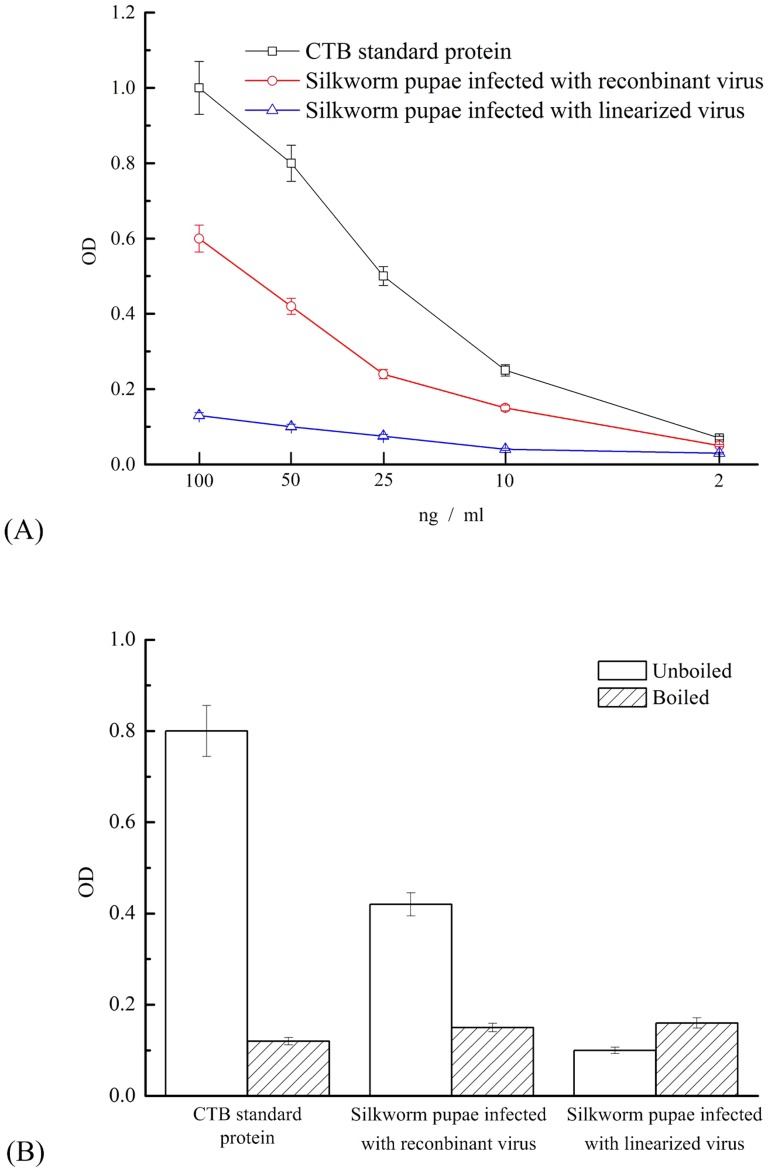
Determination of the affinity between the CTB-Aβ42 protein and GM1 by GM1–ELISA. (**A**) CTB-Aβ42 fused protein derived from silkworm pupae infected with recombinant virus (circles), positive control, CTB standard protein samples (squares) and negative control, silkworm pupae infected with linearized virus(triangles). (**B**) The three mentioned samples, both boiled and non-boiled, with the same amounts (50 ng CTB/ml) were measured for A492 signal levels. The results are presented as the means ± SE.

To determine the binding affinity of the CTB-Aβ42 fusion protein derived from silkworm pupae, GM1-ELISA was performed with GM1 as the capture molecule, and the CTB standard was used to produce a standard curve. An increase in the concentration-specific absorption signal was observed in the protein-expressing silkworm pupae samples compared with the samples infected with linearized virus (Fig. 6A, B). The GM1–ELISA analysis showed that the CTB-Aβ42 fusion protein produced from silkworm pupae formed the pentamers. These results are consistent with those from the western blot.

### Anti-Aβ42 antibody production in B6C3-Tg mice

**Figure 7:**
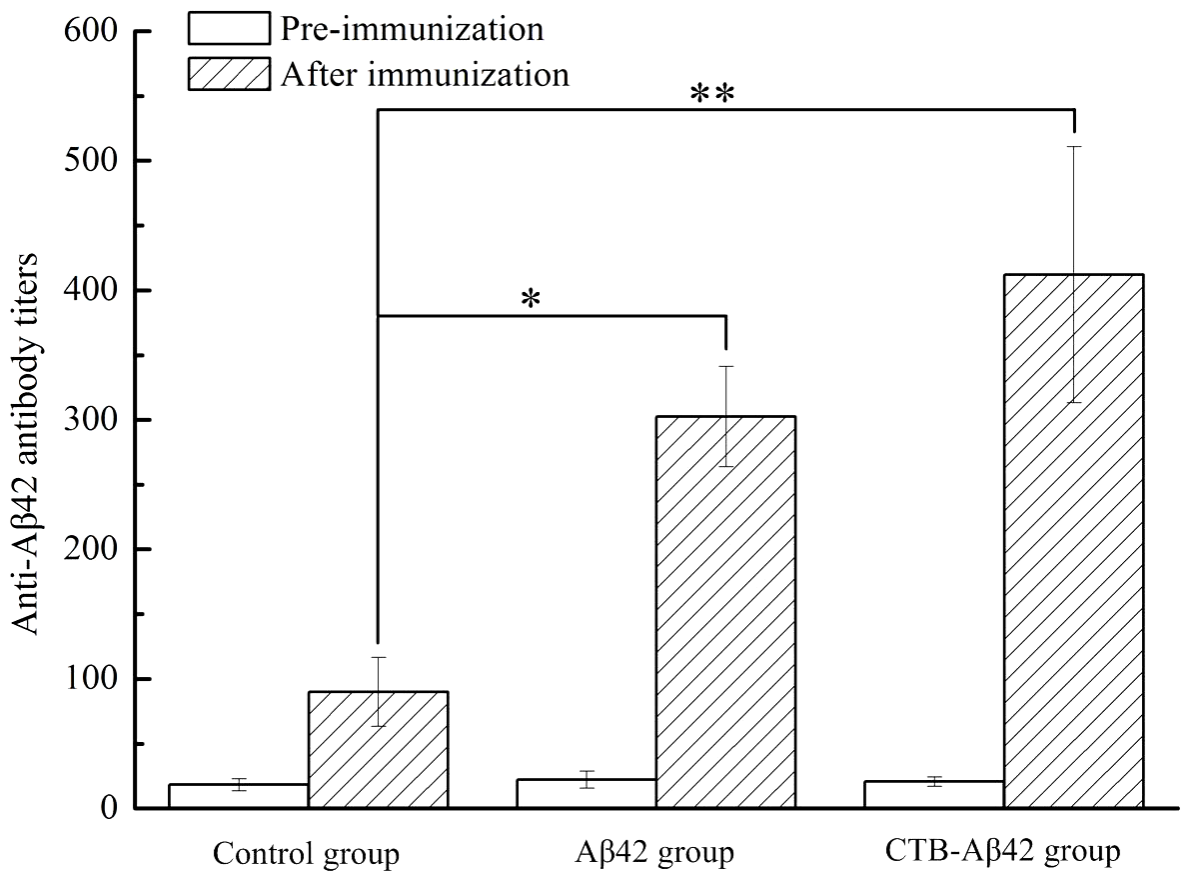
Serum anti-Aβ42 antibody titers. The results are presented as the mean titer values. * P<0.05, ** P<0.01, versus mice of control group.

We examined whether the silkworm pupae vaccination was effective in a B6C3-Tg mouse model, in which Aβ accumulates in an age-dependent manner. Oral immunization of the model mice was performed starting at 5 weeks of age and was continued for 9 months. Samples were collected when the animals reached 11 months of age. First, the anti-Aβ antibody titers were detected and calculated. Compared with the control mice treated with PBS, the anti-Aβ antibody titer was elevated significantly in two vaccine-inoculated groups of mice, and the CTB- Aβ42 group showed a higher increase in titer (Fig. 7).

#### Evaluation of the memory and cognitive ability of vaccinated mice using the water Morris maze

**Figure 8:**
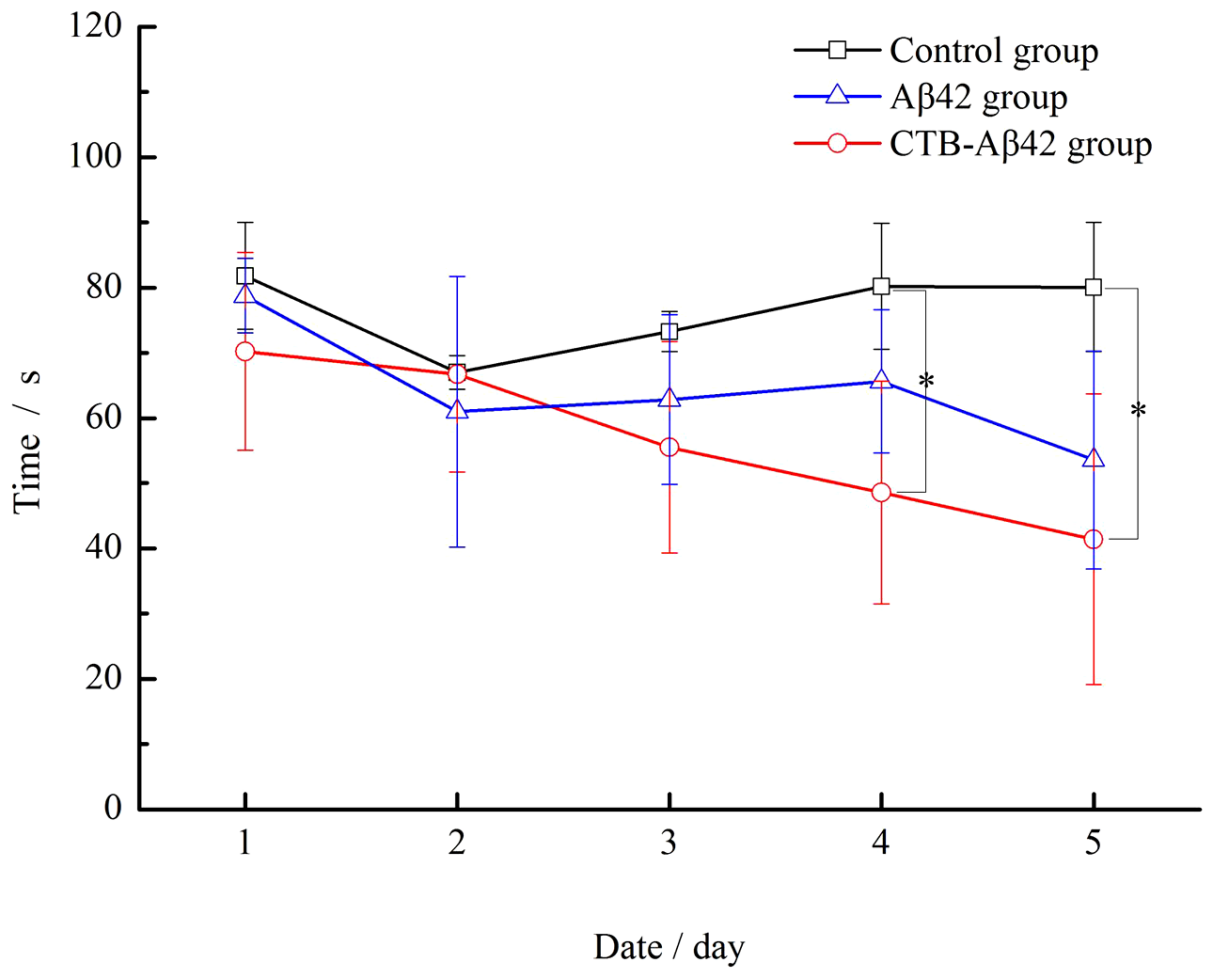
Learning curves showing the average latency (mean ± SE) to find the platform (days 1–5).

Probe trial test: To investigate effects on memory and improved cognitive ability after silkworm pupae vaccination, the ability of the transgenic AD model mice to acquire, process, and recall spatial information was assessed in the water Morris maze test using escape latency as a learning indicator. In contrast to the control group, the performance of the two vaccinated groups significantly improved over the 5 training days (Fig. 8). The difference between the groups was significant (P<0.05) on the fourth and fifth days. The escape latency was significantly shorter for the CTB-Aβ42-vaccinated mice as opposed to the control group (*P*<0.05), whereas the Aβ42-vaccinated mice showed no significant difference compared with the control group (*P*>0.05) or the CTB-Aβ42-vaccinated group (*P*>0.05). The respective asymptotic levels on the fourth day were 80.2±27.41 s (mean ± SD) for the control group, 66.89±30.07 s (mean ± SD) for the Aβ42-vaccinated group, and 48.59±30.9 s (mean ± SD) for the CTB-Aβ42-vaccinated group.

**Figure 9:**
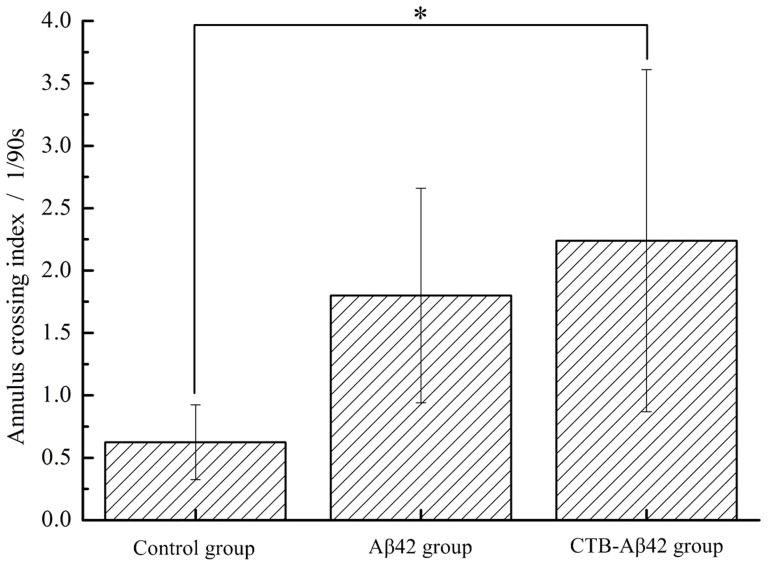
Annulus crossing index during the probe trial after the last training trial on the 6^th^ day. * P<0.05, versus mice of control group.

Place navigation test: On the sixth day, the mice were allowed to search for the platform from every quadrant for 90 s in an empty maze. The CTB-Aβ42-vaccinated group and the Aβ42-vaccinated group spent more time in the target quadrant (TQ) than did the control group. The number of crossings over the exact former location of the platform, a more refined parameter for the spatial bias in the maze, was also recorded. These measurements showed that the CTB-Aβ42-vaccinated group crossed the former location of the platform significantly more often than did the control group (*P*<0.05). The Aβ42-vaccinated group showed no significant difference from the control group (Fig. 9).

### Immunohistochemical analysis of B6C3-Tg mouse brains

**Figure 10:**
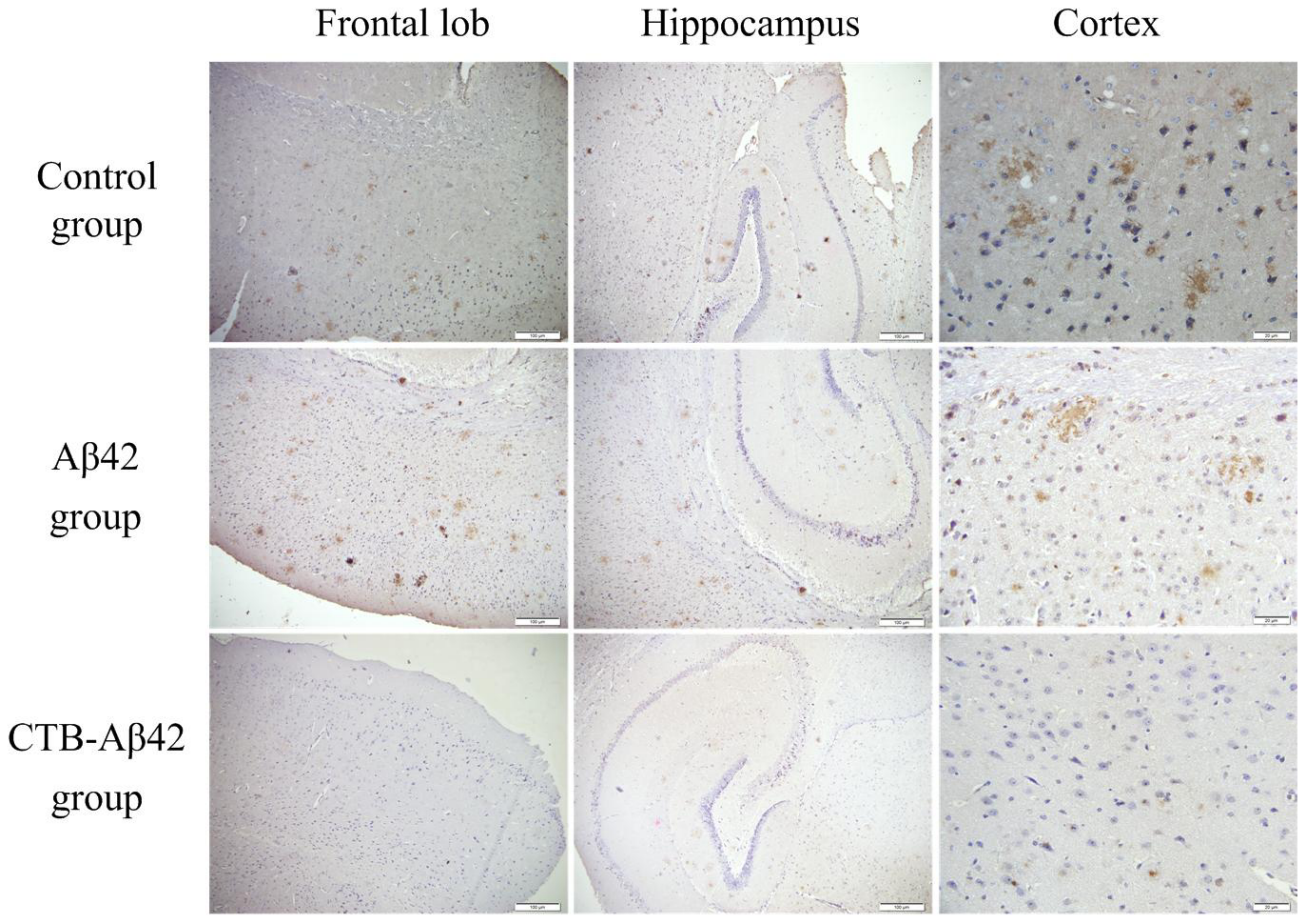
Immunostaining of B6C3-Tg mouse brains. Left, frontal lobe (Bar  = 100 µm); middle, hippocampus (Bar  = 100 µm); right, cortex (Bar  = 20 µm).

To investigate amyloid-β burden in the brain, one hemisphere of each B6C3-Tg mouse brain was immunostained with polyclonal anti-beta amyloid antibody (Abcam, Cambridge, UK). Compared with the control group mice, Aβ and CTB-Aβ42-fed mice displayed a reduced amyloid-β content in the frontal lobe, hippocampus and cortex (Fig. 10). We observed that brain tissues from the mice of the control group or the Aβ42 group developed large amyloid plaques in the frontal lobe, hippocampus and cortex. However, brain tissues from the mice of the CTB-Aβ42 group showed small and isolated amyloid plaques in the frontal lobe, hippocampus and cortex. Quantitative immunohistochemistry showed a significant decrease in the percent area of immunoreactivity to Aβ antibodies in the brain of the two vaccinated groups compared to control group mice (Aβ42-fed group *p*<0.05 and CTB-Aβ42-fed group *p*<0.01) (Table 2). The silkworm pupae vaccination with CTB-Aβ42 therefore exhibits a very pronounced effect on the prevention of Aβ deposition.

**Table 2:** Quantitative image analysis for immunoreactivity of Aβ in brains of all B6C3-Tg transgenic mice.

Groups	Control	Aβ42	CTB-Aβ42
% area	2.16±0.60	1.15±0.52*	0.50±0.20**

* P<0.05,** P<0.01, versus mice of control group.

#### ELISA test for Aβ42 peptides in mouse brains

**Figure 11:**
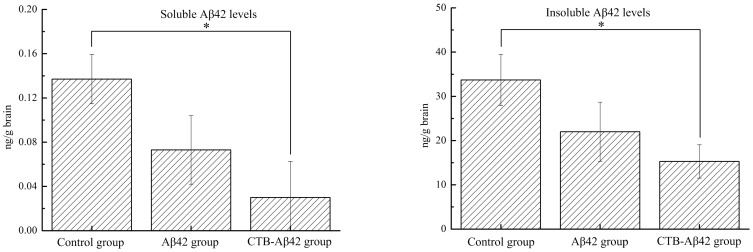
Levels of Aβ42 peptides in the brain. * P<0.05, versus mice of control group.

To confirm the ability of our vaccine to decrease the levels of Aβ42 peptides in the brain of the AD model mice, a sandwich ELISA assay, specific for soluble and insoluble Aβ42, was performed on one hemisphere of the brains from each group of mice. The Aβ42 level (from both the soluble and Tris-insoluble fractions in the brain) was significantly lower in the CTB-Aβ42-fed group than in the control group. However, the levels of Aβ42 in the Aβ42-fed group, although low, were not significantly different from those of the control mice (Fig. 11).

## Discussion

The silkworm baculovirus expression system is a high-efficiency expression system that is suited for expression of recombinant proteins. Since Chenjian et al. first used silkworm pupae to express a human biomedical protein (human granulocyte-macrophage colony-stimulating factor) [16], several biomedical proteins have been successfully expressed in silkworm pupae. For example, we have produced an H5N1 influenza vaccine [17] using this technique. Compared with the silkworm larvae, pupae have many advantages. First of all, as a byproduct of the silk reeling industry, silkworm pupae are low-cost and commercially available from farmers. The utilization of this byproduct turns “waste” into wealth. Secondly, silkworm pupae can remain active for a long time at cooler temperatures [16]. Therefore, silkworm pupae may be more convenient and economical for the expression of heterologous proteins than insect cells and silkworm larvae.

We aim to develop an edible vaccine therapy for AD. In edible vaccines, antigens enter the body through the mucosal epidermis, making the method safer and milder than hypodermic injection. Jun Nojima et al. administered green pepper leaves expressing GFP-Aβ mixed with CTB into transgenic AD model mice, and anti-Aβ antibodies were induced and a reduction of Aβ in the immunized mouse was observed after oral vaccination [18]. Rika Ishii-Katsuno et al. administered rice expressing GFP-Aβ mixed with CTB to Tg2576 AD model mice, and they observed an induction in anti-Aβ antibodies and a decreased level of intracerebral Aβ [19]. These two studies proved that transgenic plant vaccines have therapeutic effects on the mouse model of AD. Our strategy is to develop a silkworm pupae vaccine expressing a fusion protein of CTB and Aβ42. Silkworm pupae can conveniently and quickly be made into freeze-dried powder, and the activity of the specific protein is retained during long-term, room temperature preservation (although low temperature preservation is better). As a food product, silkworm pupae have been regarded as a new type of high quality protein; they contain all of the amino acids the human body needs and can be used as a dietary supplement or food additive [20]. In our previous work, we showed that freeze-dried silkworm pupae powder expressing specific proteins can be taken directly to prevent disease, such as avian influenza [17],highlighting this product's preventative and therapeutic effects, safety and lack of side effects.

CTB is a good immune adjuvant and conveying carrier antigen, and, after coupling or when expressed as a fusion protein, it can enhance the adjuvant and carrier effect of a target peptide, inducing the body to produce resistance and a systemic immune response to the specific antigen. To test whether CTB could be an effective carrier protein, Arati Limaye et al. linked CTB and the GFP gene to the poria cocos enzyme gene and expressed these CTB and GFP fusions in the tobacco chloroplast. After feeding the tobacco to mice, the researchers performed a detailed analysis of the physiological and biochemical characteristics of the mice. The authors found that the CTB-GFP fusion protein can be absorbed by intestinal epithelial cells and intestinal lymphoid tissue. Pentamers of the fusion protein could combine on the GM1 receptor on the cell membrane surface and were subsequently taken up into the phagosome. The fusion protein then traveled to the endoplasmic reticulum, where it was cut by the ubiquitous intracellular Furin enzyme, separating the CTB and GFP proteins. CTB, still associated with the GM1 receptor, was transported to the cells of the basal side, while the GFP molecules were exported via the golgi apparatus and entered the lymphatic circulation before working their way into the circulatory system [21].

Amyloid plaque and neurofibrillary tangles (NFTs) are cardinal pathological characteristics of Alzheimer's disease. There is a neuropathological analysis of 3 patients with AD subjected to the clinical trial of an Aβ peptide vaccine (AN1792). The results clearly showed reduced amyloid plaques and astrogliosis, whereas neurofibrillary tangles and deposits of aggregated amyloid in the blood vessels remained [6]. These findings are very similar to the phenomenon observed after AD model mice were immunized, indicating that immunotherapy can effectively remove Aβ plaques in human patients [22]. Autoimmunity, an issue of concern regarding Aβ-targeted vaccination, may occur in humans; nevertheless, it has not been found in vaccinated APP mice. Autoimmunity is an acquired immune response to self-antigens, and tissue damage is caused by autoimmune diseases. A significant difference in the immune response to self-antigens may be found between mice and humans. Mice are less likely to acquire autoimmune diseases than humans because humans only express human Aβ [23]. Meningoencephalitis that occurred in the AN1792 clinical trial is thought to be related to the T-cell mediated autoimmune response [24]. Production of the Alzheimer's vaccine emphasizes inducing humoral immunity mediated by the T helper (Th) type 2 cell (B cell) while eliminating potential cellular immunity mediated by Th1 cells.

To induce the Th2-polarized immune response, some teams used the Th2-type adjuvants, such as interleukin-4 [25], Alum [26], mannan [27], the cholera toxin B subunit [10], [11], [19], *Escherichia coli* enterotoxin [28], and mucosal vaccination [29]. Many studies on animals have shown that oral or nasal feeding of an antigen fused to CTB is safe and effective, and it can induce vigorous humoral immunity but suppress cellular immunity [24].

Our silkworm pupae vaccines expressing both Aβ42 and CTB-Aβ42 decreased the levels of soluble and insoluble Aβ42 in the vaccinated AD model mice and decreased the extent of the amyloid-β burden (senile plaques) in the brain. In the water Morris maze test, the vaccinated AD model mice showed significantly improved cognition (the ability to acquire, process, and recall spatial information). In all assays, the silkworm pupae vaccine expressing CTB-Aβ42 yielded better performance than that expressing Aβ42 alone. We did not detect any side effects from the vaccine.

Over the course of our vaccination, we immunized each mouse with 6 µg of Aβ42 antigen, and the total intake of silkworm pupae was 12 g per mouse. If this was converted to a human dose, only 2-3 g of silkworm pupae vaccines would be needed to prevent Alzheimer's disease. Capsule-type drugs made of silkworm pupae are very convenient, and the technology is proven. The capsule-type drug of hGM-CSF made from silkworm pupae has completed phase II clinical trials. Silkworm pupae have been determined to be safe as oral drug carriers. The new oral vaccine may be better tolerated than the existing injected vaccination with adjuvant in human patients. Because no model mice can fully express the neuropathology features of AD, the preventative effect we made in the model mice requires further verification using primates and clinical trials in humans. Our results suggest that the silkworm pupae vaccine expressed CTB-Aβ42 provides the foundation for novel and promising Alzheimer's vaccination strategy.
